# The importance of a taste. A comparative study on wild food plant consumption in twenty-one local communities in Italy

**DOI:** 10.1186/1746-4269-3-22

**Published:** 2007-05-04

**Authors:** Maria Pia Ghirardini, Marco Carli, Nicola del Vecchio, Ariele Rovati, Ottavia Cova, Francesco Valigi, Gaia Agnetti, Martina Macconi, Daniela Adamo, Mario Traina, Francesco Laudini, Ilaria Marcheselli, Nicolò Caruso, Tiziano Gedda, Fabio Donati, Alessandro Marzadro, Paola Russi, Caterina Spaggiari, Marcella Bianco, Riccardo Binda, Elisa Barattieri, Alice Tognacci, Martina Girardo, Luca Vaschetti, Piero Caprino, Erika Sesti, Giorgia Andreozzi, Erika Coletto, Gabriele Belzer, Andrea Pieroni

**Affiliations:** 1 School of Life Sciences, University of Bradford, Richmond Bd., Richmond Rd., Bradford, BD71DP, Bradford, UK; 2 University of Gastronomic Sciences, P.za Vittorio Emanuele 9, I-12060 Bra/Pollenzo, Italy; 3 SCH Group, Department of Social Sciences, University of Wageningen, Postbus 8060, NL-6700 DA Wageningen, The Netherlands

## Abstract

A comparative food ethnobotanical study was carried out in twenty-one local communities in Italy, fourteen of which were located in Northern Italy, one in Central Italy, one in Sardinia, and four in Southern Italy. 549 informants were asked to name and describe food uses of wild botanicals they currently gather and consume. Data showed that gathering, processing and consuming wild food plants are still important activities in all the selected areas. A few botanicals were quoted and cited in multiple areas, demonstrating that there are ethnobotanical contact points among the various Italian regions (*Asparagus acutifolius, Reichardia picroides, Cichorium intybus, Foeniculum vulgare, Sambucus nigra, Silene vulgaris, Taraxacum officinale, Urtica dioica, Sonchus and Valerianella *spp.). One taxon (*Borago officinalis*) in particular was found to be among the most quoted taxa in both the Southern and the Northern Italian sites.

However, when we took into account data regarding the fifteen most quoted taxa in each site and compared and statistically analysed these, we observed that there were a few differences in the gathering and consumption of wild food plants between Northern and Southern Italy. In the North, Rosaceae species prevailed, whereas in the South, taxa belonging to the Asteraceae, Brassicaceae, and Liliaceae s.l. families were most frequently cited. We proposed the hypothesis that these differences may be due to the likelihood that in Southern Italy the erosion of TK on wild vegetables is taking place more slowly, and also to the likelihood that Southern Italians' have a higher appreciation of wild vegetables that have a strong and bitter taste.

A correspondence analysis confirmed that the differences in the frequencies of quotation of wild plants within the Northern and the Southern Italian sites could be ascribed only partially to ethnic/cultural issues. An additional factor could be recent socio-economic shifts, which may be having a continued effort on people's knowledge of wild food plants and the way they use them.

Finally, after having compared the collected data with the most important international and national food ethnobotanical databases that focus on wild edible plants, we pointed out a few uncommon plant food uses (e.g. *Celtis aetnensis *fruits, *Cicerbita alpine *shoots, *Helichrysum italicum *leaves, *Lonicera caprifolium *fruits, *Symphytum officinale *leaves), which are new, or have thus far been recorded only rarely.

## Background

In recent years, wild food plants have increasingly became the focus of many ethnobotanists in Europe. There are several reasons for this: the renewed interest in local traditional foods and neglected plant food sources [1]; the related concepts of *terroir *[2] and intangible cultural heritage [3]; and the potential of these foods as nutracauticals, and in the prevention of cancer and Ageing Related Diseases (ARDs) [4]. While in recent years an increasing number of studies and reviews have recorded food ethnobotanical knowledge in Italy [5-11] and in Europe and Turkey [12-20], very few works have tried to compare data on wild food plant gathering and consumption among contiguous areas/cultural groups [21-24], and to understand than how these phenomena change over time and space.

Food ethnobotany of wild species is currently at the crossroad of two divergent processes in Italy, and probably in other Western countries, too. These processes are: a). the erosion of Traditional Knowledge (TK), which is occurring even in the most "isolated" rural areas, where generally only the elderly people have retained this knowledge and are still accustomed to gathering and cooking wild plants; and b). the contemporaneous increase of interest in local plant food sources and neglected botanicals among the young or middle-aged most acculturated urban classes.

Clues that lead to the understanding of how knowledge and practices of gathering wild foods change over time and space, and how the cultural importance of wild food plants is shaped within a given community are crucial for answering scientific questions regarding the mechanisms of transmission of TK, and the influence social factors may have in the persistence of gathering practices, as well as the appreciation of food botanicals.

The aims of the present work were the following:

• to carry out an ethnobotanical survey on wild food plants in twenty-one selected areas in Italy, using the same methodological frameworks in each area;

• to compare the data collected in these areas, taking into consideration a few other food ethnobotanical studies that our research groups have carried out in the last ten years;

• to compare the overall data with Italian and international food ethnobotanical literature;

• to discuss if and how hypothetical differences can be attributed to environmental, cultural, or social factors.

## Methods

**Figure 1:**
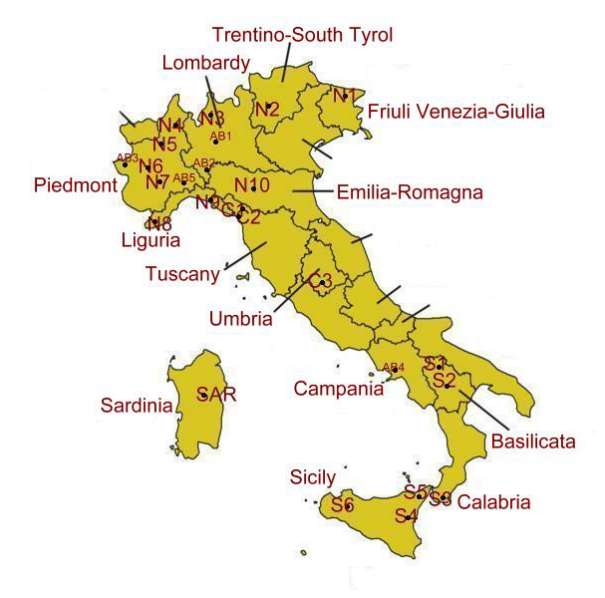
Location of the selected study areas.

Twenty-one small communities were selected in Italy: fourteen in Northern Italy, one in Central Italy, one in Sardinia, and four in Southern Italy (Table 1 and Figure 1). Each of these communities was represented by one or more villages located within homogenous mountainous, rural or even peri-urban areas. The considered areas included a broad variety of ecological and socio-economic environments (Table 1).

**Table 1:** List of the all selected study areas, including those (in italics) that have been the object of previous studies (see Methods) and have been considered here for comparative purposes only.

*Community/area code*	*Community/area name*	*Region*	*Ecological and economic characteristic of the community/area*	*Ethnicity/Language*	*Number of interviewees*
N1	Val Canale/Kanatal and Carnia	Friuli-Venezia Giulia	Mountainous/alpine area: small-scale agriculture, tourism	German, Slovenian, and Friulan	25
N2	Val Lagarina	Trentino-South Tyrol	Mountainous/pre-alpine area: tourism, agriculture, industrial activities	Northern Italian	25
N3	Alta Valsassina	Lombardy	Mountainous/pre-alpine area: tourism, cow breeding, intensive industrial activities nearby	Northern Italian	25
N4	Val Grande and Verbania/Countryside	Piedmont	Mountainous/pre-alpine area: tourism	Northern Italian	25
N5	Valchiusella	Piedmont	Mountainous/pre-alpine area: eco-tourism, industrial areas close by	Northern Italian	25
N6	Moncalieri and Ternavasso Lake area	Piedmont	rural/industrialised area: intensive agriculture (vineyards), industrial activities	Northern Italian	47
N7	Verduno	Piedmont	Rural/industrialised area: intensive agriculture (vineyards), eno-gastronomic tourism, minor industrial activities	Northern Italian	23
N8	Val Nervia	Liguria	rural area: intensive agriculture (flowers, olive trees, vineyards); thermal tourism near by	Northern Italian	25
N9	Chiavari hills	Liguria	Rural area: tourist activities near by	Northern Italian	28
N10	Quattro Castella	Emilia-Romagna	Rural/industrialised area: intensive agriculture, pig breeding, food industries	Northern Italian	25
C1	Massa Carrara/Countryside	Tuscany	rural area: agriculture (olive trees), industrial activities nearby	Central Italian	25
*C2*	*Garfagnana*	*Tuscany*	*Mountainous/rural area: tourism, small-scale agriculture, minor industrial activities*	*Central Italian*	*95*
C3	Terni/Contryside	Umbria	rural area: agriculture (olive trees and vineyards); industrial activities nearby	Central Italian	27
*S1*	*Ginestra*	*Basilicata*	*Rural area: agriculture (vineyards and olive trees), industrial activities nearby*	*Albanian*	*68*
*S2*	*Castelmezzano*	*Basilicata*	*Rural area: small scale agriculture, tourism*	*South Italian*	*86*
*S3*	*Gallicianò*	*Calabria*	*Rural area: small scale agriculture*	*Greek*	*36*
S4	Pisano Etneo	Sicily	Rural area: small scale agriculture, industrial activities nearby	South Italian	21
S5	Messina/Countryside	Sicily	Rural/industrialised peri-urban area: small scale agriculture, industrial activities nearby	South Italian	25
S6	Alcamo area	Sicily	rural area: agriculture (olive trees and vineyards), eno-gastronomic tourism	South Italian	25
SAR	Dorgali, Oliena, and Gavoi	Sardinia	Rural area: small scale agriculture (olive trees), minor industrial activities nearby	Sardinian	25
AB1	Milan/Hinterland	Lombardy	Industrialised peri-urban area	Northern Italian	24
AB2	Valverde	Lombardy	Rural area: small scale agriculture and food industries	Northern Italian	23
AB3	Val Sangone and Susa Valley	Piedmont	Mountainous/pre-alpine area: tourism	Northern Italian and Franco-Provençal	26
AB4	Naples/Countryside	Campania	Rural/industrialised peri-urban area: intensive agriculture, industrial activities nearby	Southern Italian	25
AB5	Trisobbio	Piedmont	Rural area: intensive agriculture (vineyards)	Northern Italian	30

Interviews were conducted during the winter, spring, and summer of 2006, with approximately twenty-five informants in each community (total number of interviewees: 549). The informants were selected using snowball techniques and preference was given to those community members *emically *considered to be "knowledgeable persons" in the field of wild food plant cuisine.

In the definition of *food plants *we excluded herbal teas, which are generally ingested in Italy for obtaining a healing activity or as preventive medicine. They are very rarely consumed within proper "food contexts". We included instead plants used for preparing digestive liqueurs, since these are often perceived as being the final part of a "meal".

Informants were asked to name wild and semi-domesticated food plants they knew and used, and to precisely describe their culinary processing. We expressly decided not to focus on "traditional uses" alone, since we were interested in gathering information on the *actual use *of wild food plants, including recently evolved "new" food plant uses, even if these have arisen from modern media and popular books.

A clear expression of consent was also obtained before each interview. Throughout this field study, the ethical guidelines adopted by the ICE/International Society of Ethnobiology [25] and Italian Association of Ethno-Anthropologists (AISEA [26]) were rigorously observed.

During the interviews, notes were taken, and whenever possible audio or video recordings were made with the permission of the interviewees.

If they were available, plants quoted during the interviews were gathered, and deposited in twenty-one small herbaria. Identification was carried out using the *Flora d'Italia *[27]. The ethnobotanical data were analysed and compared with data that we had collected in four previous field studies that had been carried out by our research group during the last ten years in Northern Tuscany, Basilicata, and Calabria (the last study being performed in the field by Dr Sabine Nebel, Zurich) [6,9,10,28].

Statistical analysis was carried out using the software, NTSYSpc Version 2.0 (Exeter Software) [29], in a way analogous to that used by other scholars processing ethnobiological data regarding home gardens [30]. The data were also compared with the most recent review of Italian ethnobotany [31], and with the most important international databanks on wild food plants [32-38].

## Results and discussion

### Most cited taxa

In Table 2 we presented a summary of the fifteen most commonly quoted taxa in all selected study areas, and in those analysed in the aforementioned four previous studies carried out over the last ten years.

**Table 2:** List of all fifteen most quoted taxa recorded in all the considered areas. In bold are the eleven taxa that were quoted in at least eight of the considered study areas. In italics are the area codes that refer to sites that were investigated in previous ethnobotanical studies by our research group.

Botanical taxa	English common names and part uses	N1 n = 25	N2 n = 25	N3 n = 25	N4 n = 25	N5 n = 25	N6 n = 47	N7 n = 23	N8 n = 25	N9 n = 28	N10 n = 25	C1 n = 25	*C2 n = 95*	C3 n = 27	*S1 n = 68*	*S2 n = 86*	*S3 n = 36*	S4 n = 21	S5 n = 25	S6 n = 25	SAR n = 25
*Allium ursinum *L.	Bear's garlic, leaves	+																			
*Amaranthus retroflexus *L.	Pigweed, leaves														+				+		
*Apium nodiflorum *(L.) Lag.	Fool's watercress, aerial parts												+								
*Arbutus unedo *L.	Strawberry tree, fruits																				+
*Aruncus dioicus *(Walt.) Fernand	Goat's beard, shoots		+	+		+	+														
***Asparagus acutifolius *L.**	**Wild asparagus, shoots (1)**	+			+						+	+		+	+	+			+	+	+
*Bellis perennis *L.	Daisy, whorls							+													
*Beta vulgaris *L. ssp. *maritima *(L.) Arcang.	Wild beet, leaves								+	+	+							+		+	+
***Borago officinalis *L.**	**Borage, aerial parts (2)**				+	+	+	+	+	+			+	+	+	+		+	+	+	+
*Brassica fruticulosa *Cyr.	Wild mustard, leaves																	+		+	
*Calamintha nepeta *Savi	Lesser calamint, leaves												+								
*Campanula rapunculus *L.	Rampion, whorls and roots			+						+			+	+							
*Capparis spinosa *L.	Caper, flower buds and fruits								+										+		
*Capsella bursa-pastoris *(L.) Med.	Shepherd's purse, whorls				+																
*Carum carvi *L.	Caraway, fruits	+																			
*Celtis aetnensis *(Tornabene) Strobl	Etnean hackberry tree, fruits																	+			
*Centranthus ruber *(L). DC.	Red valerian, whorls								+												
*Chenopodium album *L.	Fat hen, leaves														+				+		
*Chenpodium bonus-henricus *L.	Good King Henry, leaves	+	+			+															
*Chondrilla juncea *L.	Naked weed, whorls and shoots														+		+				
*Cicerbita alpina *(L.) Wallr.	Alpine thistle, whorls	+	+																		
***Cichorium intybus *L.**	**Wild chicory, whorls and shoots (3)**	+										+	+	+	+	+	+	+	+	+	+
*Clematis vitalba *L.	Traveller's joy, shoots										+		+	+	+	+					
*Chrisanthemum segetum *L.	Corn-marigold, whorls																				+
*Cornus mas *L.	Cornelian cherry tree, fruits		+			+					+										
*Crataegus *spp.	Hawthorn, fruits		+																		
*Crithmum maritimum *L.	Rock samphire, young aerial parts								+												
*Crepis *spp.	Hawksbeard, whorls										+		+			+					
*Cynara cardunculus *L. ssp. *cardunculus*	Wild artichoke, flower receptacles, stems, and roots																+				
*Daucus carota *L.	Wild carrot, whorls								+										+		
*Diplotaxis tenuifolia *L.	Wall rocket, leaves	+									+										
***Foeniculum vulgare *L.**	**Wild fennel, young aerial parts and fruits (4)**								+	+		+		+	+	+		+	+	+	+
*Fragaria vesca *L.	Wild strawberry, fruits			+			+														
*Galactites tomentosa *Moench.	Galactites, leaves and stems																				+
*Hedypnois cretica *Willd.	Cretan weed, whorls																+				
*Hirschfeldia incana *(L.) Lagr.-Foss.	Hoary mustard, leaves																+		+		
*Humulus lupulus *L.	Wild hop, shoots		+			+	+	+	+												
*Hyoseris radiata *L.	Hyoseris, whorls									+											
*Hypochoeris *spp.	Cat's ear, whorls			+								+	+				+	+			
*Juniperus communis *L.	Juniper, fruits	+	+								+			+							
*Lactuca *spp.	Wild lettuce, whorls														+			+			
*Leontodon *spp.	Hawkbit, whorls			+							+	+				+					
*Leopoldia comosa *Parl.	Tassel hyacinth, bulbs														+	+					
*Malva sylvestris *L.	Mallow, young leaves																			+	
*Myrtus communis *L.	Myrtle, fruits																		+		+
*Nasturtium officinale *R. Br.	Watercress, aerial parts			+	+	+					+	+								+	+
*Origanum heracleoticum *L.	Sicilian oregano, flowering tops															+	+				
*Origanum vulgare *L.	Oregano, flowering tops	+								+											
*Papaver rhoeas *L.	Corn poppy, whorls		+					+		+		+			+	+					
*Parietaria officinalis *L.	Pellitory of the wall, leaves							+				+									
*Phyteuma *spp.	Rampion, whorls and roots					+															
*Picris echioides *L.	Ox-tongue, whorls												+			+					
*Pinus pinea *L.	Italian stone pine, seeds								+												
*Pistacia lentiscus *L.	Mastix tree, fruits																				+
*Plantago lanceolata *L.	Plantain, young leaves							+	+		+	+									
*Polygonum bistorta *L.	Bistort, leaves					+															
*Portulaca oleracea *L.	Purslane, young aerial parts																	+	+		
*Primula vulgaris *Hudson	Primrose, whorls				+	+		+													
*Prunus spinosa *L.	Sloe, fruits										+										
*Quercus *spp.	Oak tree, fruits																				+
*Ranunculus ficaria *L.	Lesser celandine, young leaves.				+																
*Raphanus raphanistrum *L.	Wild radish, young leaves																	+	+	+	+
***Reichardia picroides *L.**	**French scorzanera, whorls (5)**								+	+		+		+		+	+	+	+		
*Reseda alba *L.	White mignonette, whorls																+				
*Robinia pseudoacacia *L.	Black locust, flowers					+		+													
*Rosa canina *L.	Dog rose, fruits		+		+		+				+		+								+
*Rubus fruticosus *L.	Blackberry, shoots and fruits	+		+	+		+		+								+				
*Rubus ideaus *L.	Raspberry, fruits			+	+																
*Rumex acetosella *L.	Sorrel, leaves		+	+			+														
*Rumex crispus *L.	Dock, leaves											+									
*Ruscus aculeatus *L.	Butcher's broom, shoots															+				+	+
***Sambucus nigra *L.**	**Elderberry tree, fruits (6)**	+	+	+	+	+	+			+	+										
*Sanguisorba minor *L.	Great burnet, leaves							+				+		+							
*Satureja montana *L.	Wild savory, leaves							+		+											
*Scolymus hispanicus *L.	Spanish salsify, midribs														+		+				
***Silene vulgaris *Moench.**	**Bladder campion, young aerial parts (7)**	+	+	+		+	+						+	+				+			
*Sinapis *spp.	Wild mustard, leaves														+	+	+		+	+	
***Sonchus *spp.**	**Thistle, whorls (8)**								+	+		+	+	+		+	+	+	+	+	+
*Sisysmbrium officinale *(L.) Scop.	Hedge mustard, leaves														+						
***Taraxacum officinale *Weber**	**Dandelion, leaves (9)**	+	+	+	+	+	+	+	+	+		+	+				+				
*Tordylium apulum *L.	Roman pimpernel, leaves														+						
*Thymus serpyllum *s.l. L.	Wild thyme, leaves			+						+				+							
*Tragopogon pratensis *L.	Goat's beard, aerial parts (including flower buds)					+	+	+	+												
*Urospermum *spp.	Sheep's beard, whorls									+							+				
***Urtica dioica *L.**	**Nettle, leaves (10)**	+	+		+	+	+	+	+	+	+	+	+								
*Vaccinium myrtillus *L.	Bilberry, fruits	+		+	+																
***Valerianella *spp.**	**Corn salad, whorls (11)**		+	+	+		+	+			+		+								
*Viola odorata *L.	Violet, leaves				+	+	+	+													

We excluded from this analysis five sites (indicated in Table 1 and Figure 1 as "AB", for "aborted"), since the ethnobotanical data collected there were very restricted (less than twenty quoted wild food taxa). Data collected in these sites were only analysed in relation to uncommon food plant uses that have evolved in recent times (see following section).

**Figure 2:**
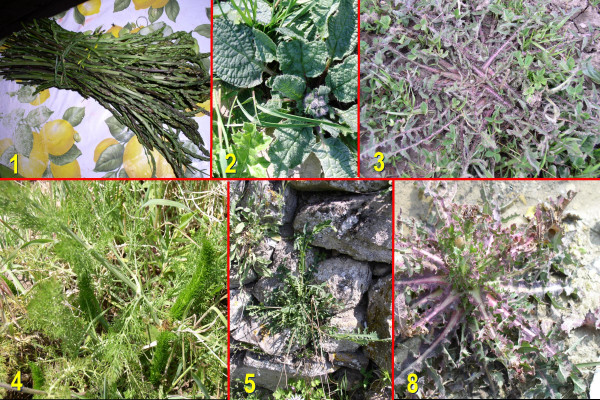
A few of the most commonly quoted wild vegetables in the study areas. Numbers refer to those in the second column of the list of plants reported in Table 2.

In Table 2 we showed in bold the taxa (eleven) that have been among the most quoted in at least eight of the considered communities (Figure 2): *Asparagus acutifolius, Reichardia picroides, Cichorium intybus, Foeniculum vulagre, Sambucus nigra, Silene vulgaris, Taraxacum officinale, Urtica dioica, Sonchus and Valerianella *spp.).

### Comparative analysis

**Figure 3:**
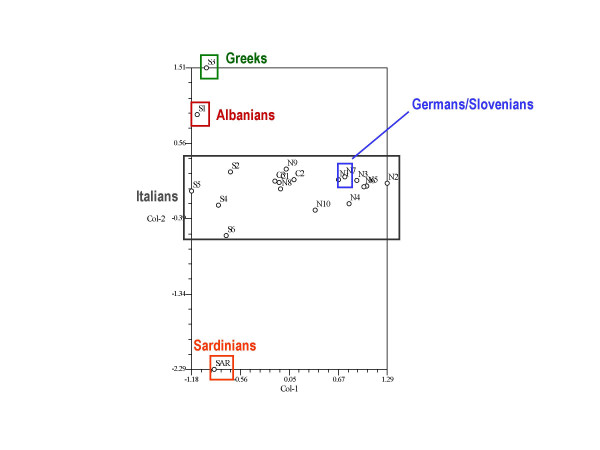
Correspondence analysis carried out on the food ethnobotanical uses of the fifteen most cited taxa among all the considered sites.

In Figure 3 we used the same data as in Table 2 in a correspondence analysis, after having built a matrix in which we took into account for the each site the presence/absence of the selected fifteen most cited taxa. Figure 3 shows how the sites characterized by ethnic minority groups (non-Italians) – except for the German and Slovenian communities of Carnia and Val Canale/Kanaltal – appear to be quite distant from the location of the Italian sites in the diagram. This could confirm, of course, that these communities are accustomed to gathering and consuming a few unusual wild plants, which could also be seen being as a distinctive sign of a diverse ethno-historical origin (for example, *Reseda alba *consumption among the Greeks of Gallicianò in Calabria [9]).

However, to ascribe differences among the most quoted wild food botanicals to ethno-historical reasons alone could be making dangerous assumptions, since the environmental/ecological availability of plants must also be a crucial factor that greatly influences the plant selection criteria of local communities.

**Figure 4:**
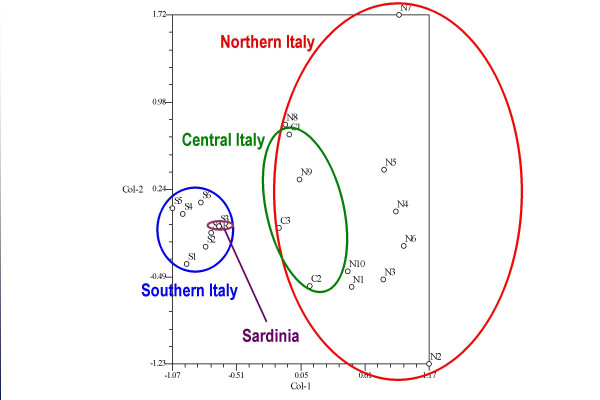
Correspondence analysis carried out on the food ethnobotanical uses of the fifteen most cited taxa among all the considered sites, after eliminating those botanical taxa that are not ecologically available in all study areas.

In order to to discriminate between the ecological and the cultural components, in Figure 4 the same statistical analysis was performed, but only after having first eliminated those taxa which, according to the Pignatti's *Flora d'Italia *[27] (in which plant availability is reported in detail for every region/area) and according to personal observation, are not widespread among *all *the studied areas.

In this way we tried to avoid having a situation where eventual differences could be exclusively attributed to differences in environmental factors/availability of the species among the diverse areas.

This aspect, which is indeed a crucial one in ethnobotanical comparative analysis, seems at times to have been underestimated in other comparative works, where authors do not appear to take serious account of the *factual ecological availability *of the cited taxa in the specific investigated sites (which in ethnobotany are always generally represented by very restricted areas/communities) [39]. Many floristic data/national and regional "Floras" in different countries are, for example, only available for very broad geographical areas, so they are not sufficiently rigorous as a basis for making assumptions on the occurrence/absence of a specific taxon within given restricted areas, which generally represent the scenario where ethnobotanical studies are actually conducted. As a consequence, referring commonalities and differences in plant usages to cultural factors only is highly questionable.

Hence, as a demonstration of what we are trying to explain, we obtained very different pictures from comparing the rough data (Figure 3) and the "corrected" data (after having eliminated data related to taxa available in a few selected areas only, Figure 4).

### Ethnicity versus acculturation

Figure 4 clearly showed that ethnicity seems not to play a major role in differentiating the communities. In fact, if we analyse sites N1 (Slovenians and Germans), S1 (Albanians), S3 (Greeks), and SAR (Sardinians), we can see in the diagram that N1 was located very close to most of the other Northern Italian sites (as it was in Figure 3), while S1, S3 and SAR were indistinguishable from the bulk of the other locations in Southern Italy. The "cultural" differences that clearly appeared in Figure 3 were cancelled out in Figure 4, probably because of the elimination of a few distinctive plant uses referring to taxa that were not ubiquitously available.

The correspondence analysis in Figure 4 showed that it was still possible to distinguish between the South Italian and Sardinian sites on the one side, and the Central and Northern Italian sites on the other. Moreover, the Northern Italian locations appeared more heterogeneous in their food ethnobotanical quotation frequencies than the Southern Italian ones.

The only really relevant shifts towards the hypothetical core centre of all the North Italian locations was shown by the sites N2 and N7, which were neither located in special areas, nor inhabited by particular ethnic groups.

For N7 in particular, we could propose the hypothesis that recent social changes, rather than ethnic or cultural issues, may be crucial for explaining its isolated position in the correspondence analysis diagram. N7 was in fact located in a highly modernized agricultural environment (with the "wine industry" and eno-gastronomic tourism being the core activities), and where the gathering of wild botanicals is mostly done on the basis of a renewed interest in alternative cooking and the sudden trendiness of speciality local foods, which have been publicized mainly by the small-scale market chains and networks promoting "typical products" (*prodotti tipici*). These phenomena have also gained considerable credence in the last few years through the activities of the Slow Food movement [40], which was born very close to this area, and which has its headquarters there.

Hence, the particular "behaviour" of the N2 site is quite difficult to explain in socio-economic terms, and neither could it be related to the isolation of the valley, since this iste is located on what was traditionally one of the most important communication, travel and trade routes between the Austrian North and Northern Italy. The N8 and N9 sites appeared in the diagram very close the Central Italian site C1, which came as not surprise, since all three were located in the hills along the Ligurian coast, and shared very similar environmental and socio-economic characteristics.

### Comparison of the most quoted food botanical families in Northern and Southern Italy: the importance of taste

**Figure 5:**
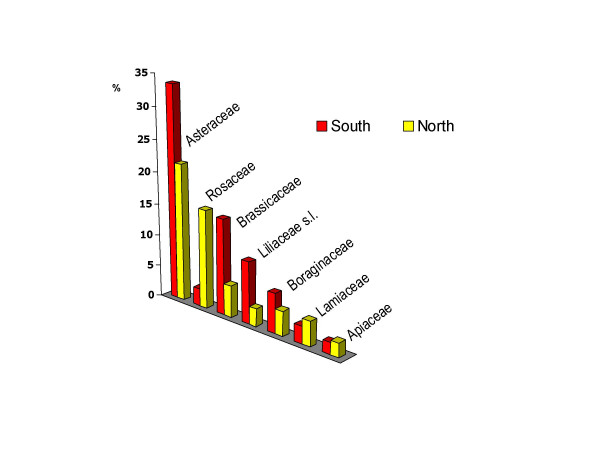
Most quoted wild food botanical families in the Northern and Southern Italian study areas.

In Figure 5 we gave a comparison between the most quoted food botanical families in the Northern and Southern Italian sites (sites C3 and SAR were allocated to the South, and sites C1 and C2 to the North).

The data was expressed as a percentage of the wild food taxa belonging to a given botanical family out of the total number of the fifteen most quoted taxa recorded in the North and the South, respectively.

It was evident from the figures that in Southern Italy Brassicaceae, Asteraceae, and Liliaceae s.l. were more often quoted as wild food plants than in Northern Italy, while for the Rosaceae we observed the opposite. It was most quoted in the North and not in the South. For other botanical families the difference did not appear to be relevant.

The fact that the frequency of quotation of most wild greens (which for the most part belong to the Asteraceae and Brassicaceae families) is remarkably higher in the Southern Italian locations could be related to a stronger persistence of traditional gathering activities of wild vegetables there, and to a higher appreciation of the bitter taste of many young Asteraceae and the particularly strong ("hot") taste of the aerial parts of a few Brassicaceae. In a previous field study that we conducted in the S1 site, we observed that these tastes (and especially the bitter one) are very popular and often associated with healthiness [10].

**Figure 6:**
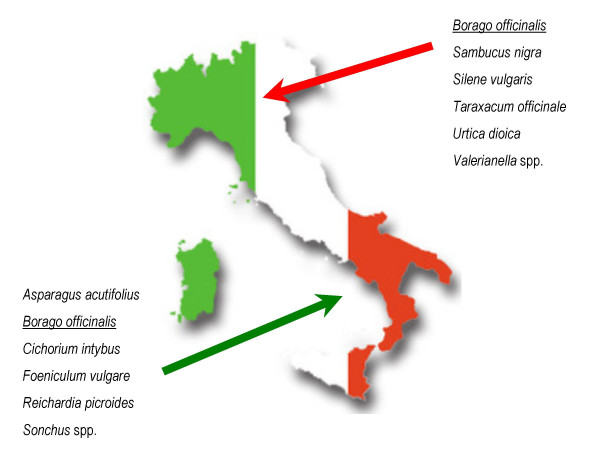
Representation of the five most quoted wild food taxa in Northern and Southern Italy (the name of the species that turned out to be among the most quoted in *both *macro-regions is underlined).

In Figure 6 we reported on the most quoted taxa in Southern and Northern Italy. One taxon (*Borago officinalis*) in particular was found to be among the most quoted taxa in the sites in both macro-regions.

### Uncommon food ethnobotanical findings

In this section we summarised a few uncommon *culinary food uses *of plants or uncommon *food plants *that we recorded during the present field studies, and that have been quoted only very rarely in the ethnobotanical literature, either in the Italian national databank [31] or in other relevant worldwide food plants databases [32-37]. We have also included in this section a few very uncommon uses of semi-domesticated taxa (or taxa that have reverted to wild status) and even cultivated taxa.

*Aruncus dioicus *(Walt.) Fernand (Rosaceae). Goat's beard. Lombardy, N3; Piedmont, N5, N6. Local name: *Barba de cavra *[N3, N6]. *Bulmit *[N5]. Wild. The young shoots are very popular in N3, N5 and N6; where they are gathered in March, boiled briefly in water, sometimes with a few aromatic plants such as sage, rosemary, oregano, and then cooked with eggs and cheese. In N5 they are also eaten raw in salads. Two previous ethnobotanical studies carried out in Piedmont [41] and in Veneto [42] mentioned that this wild vegetable is consumed in a similar way. In Friuli it is one of the ingredients in the local home-made soup based on wild greens called *pistic *[11].

*Asphodeline lutea *(L.) Reichenb. (Liliaceae). King's spear. Sicily, S6. Local name: *Musulucu*. Wild. The immature inflorescence is boiled and consumed, or utilised to aromatise a tomato sauce that is prepared for eating with noodles (in which case the inflorescence is put in the pot while the sauce is cooking, and removed before serving.). The culinary use of this taxon is so far known only in Central and Southern Italy and Sicily [5,31].

*Asphodelus albus *Miller (Liliaceae). Asphodel. Piedmont, N5. Local name: *Spars d'la bava*. Wild. The young shoots are boiled and consumed like wild asparagus. The culinary use of this taxon is so far known only in Central and Southern Italy [31].

*Brassica fruticulosa *Cyr. (Brassicaceae). Sicily, S6. Local name: Cavuliceddu. Wild. The young leaves are gathered in winter, boiled and consumed with olive oil. Sometimes they are boiled briefly, and then fried with garlic and dried tomatoes. This species seems to be very popular as a food in Sicily [5,43].

*Celtis aetnensis *(Tornabene) Strobl (Ulmaceae). Etneaen hackberry tree. Wild. Sicily, S4. Local name: *Minicuccu*. The fruits are eaten as snacks at the end of the summer, especially by children. The seeds are also used in spitting and throwing competitions, after the pulp has been chewed. This food use has thus far never been recorded in Italy.

*Centranthus ruber *(L.) DC. (Valerianaceae). Red valerian. Liguria, N9. Local name: *Cornacchia, Favoia*. Wild. The young leaves are consumed in salads, while the mature leaves are one of the diverse ingredients used in a complex wild greens-based local soup called *preboggion*. Use of this species raw in salads had previously been recorded only in Eastern Liguria [44].

*Chrysanthemum indicum *L. (Asteraceae). Garden chrysanthemum. Piedmont, N6. Local name: *Fiure di mort*. Cultivated. Petals are boiled briefly and consumed in salads with lemon, salt and pepper.

*Chrysanthemum segetum *L. (Asteraceae). Corn marigold. Sardinia, SAR. Local name: *C agarantzu masedu*. Wild. The young whorls are consumed raw in salads, and sometimes they are boiled with other wild greens. Similar uses have been recorded in previous studies in Sardinia [45] and Latium [46].

*Cicerbita alpina *(L.) Waller. (Asteraceae). Blue sow thistle. Friuli Venezia-Giulia, N1. Local name: *Radic di mont*. Wild. The young shoots are gathered at the end of the winter/beginning of the spring, they are boiled briefly in water, wine and white vinegar, and pickled in glasses, in the same liquid or in oil. After being boiled, they can be also consumed in omelettes. This use has been reported in a couple of Italian reviews on food ethnobotany or the economic botany of food plants [47,48], but never recorded in ad-hoc ethnobotanical studies.

*Dipsacus fullonum *L. (Asteraceae). Thistle. Piedmont, N4. Local name:*Artichoc*. Wild. The flower receptacles are consumed in salads, or boiled. A similar use has so far been recorded only in Sardinia [45].

*Ferula communis *L. (Apiaceae). Giant fennel. Sardinia, SAR. Local name: *Ferula*. Wild. The internal part of the stem is buried in ashes and roasted This use has been recorded in other studies in Sardinia [45].

*Galactites tometosa *(L.) Moench. (Asteraceae). Galactites. Sardinia, SAR: Local name: *Aldu biancu*. Wild. The young leaves and stems are traditionally fried in pork lard. This use has been recorded in two other studies, one in Sardinia [45] and one in Basilicata [49].

*Helichrysum italicum *L. (Asteraceae). Curry plant. Liguria, N9. Local name: *Rusmarin sarvego*. Wild. The leaves are used to aromatise various dishes. The taste is thought too be more delicate than that of rosemary. A similar use has been very recently recorded on the Tyrrhenian coast of Southern Italy [8].

*Knautia arvensis *(L.) Coulter. (Dipsacaceae). Field scabious. Piedmont, N5. Local name: *Cresta di gallo*. Wild. The young whorls are gathered in April, boiled and consumed like spinach. A food use of this taxon has been reported once in Tuscany [50].

*Lonicera caprifolium *L. (Caprifoliaceae). Italian honeysuckle. Lombardy, AB2. Local name: *Uva San Giuan*. Wild. The fruits are gathered in June and eaten raw as snacks. This use has never recorded before in Italy.

*Parietaria officinalis *L. (Urticaceae). Pellitory of the wall. Liguria, N9. Local name: *Gamba rossa*. Wild. The leaves are one of the most common ingredients of a complex wild-greens-based local soup called *preboggion*. A similar use has been recorded twice: in Piedmont [41] and Basilicata [49].

*Phyteuma *spp. (Campanulaceae). Rampion. Piedmont, N5. Local name: *Viucca*. Wild. The leaves and the roots are extremely popular in the site N5, where they are generally boiled and consumed. Similar uses were recorded in the same region [41].

*Pinus mugo *L. (Pinaceae). Dwarf mountain pine. Friuli Venezia-Giulia, N1. Local name: *Mugo*. Wild. The young shoots are put in a glass with sugar, and left in the sun for two months. The honey-like resulting product is then filtered and used as a sweetener in teas or hot milk, or a food-medicine (especially against coughs). A similar use has been recorded only once in the only previous ethnobotanical study conducted in the same region [51].

*Prunus mahaleb *L. (Rosaceae). Mahaleb cherry tree. Lombardy, AB2. Local name: *Sbosra*. Wild. The fruits are consumed raw. A similar use has been previously recorded in Abrúzzo [52] and Basilicata [49].

*Prunus laurocerasus *L. (Rosaceae). Laurel cherries. Lombardy, N3. Local name: *Sciresa de Spagna*. Semi-domesticated/reverted to a wild status. The fruits are gathered in July and consumed fresh. They are also soaked in *grappa *and eaten during the winter. A culinary use of the toxic fruits has previously been recorded in three ethnobotanical studies in Northern Italy: in Piedmont [41], Friuli [53], and Northern-Western Tuscany [28].

*Ruscus hypophyllum *L. (Liliaceae). Butcher's broom. Sicily, S6. Local name: *Sparaciu di tronu*. Wild. The young shoots are gathered in spring and boiled and consumed, or used in omelettes. To our knowledge this is the first time this plant has been mentioned for its food use.

*Ruta graveolens *L. (Rutaceae). Rue. Friuli Venezia-Giulia, N1. Local name: *Ruta*. Cultivated. Young branches of the plant are dipped in a batter, deeply fried in oil, and consumed with salt or sugar. They are also used on their own to aromatise a specific type of omelette. These peculiar food uses of rue have never been recorded in Italy.

*Santolina chamaecyparissus *L. (Asteraceae). Lavender cotton. Liguria, N9. Local name: *Erba ochetta*. Wild. Used to aromatise stewed meat and sometimes fish dishes. This culinary use of this taxon is new to Italy.

*Symphytum officinale *L. (Boraginaceae). Comfrey. Friuli Venezia-Giulia, N1. Local name: *Concuardie*. Wild. The young leaves are gathered in spring, boiled, and added to meat and old bread to make meatballs; or they are boiled and consumed fried with other greens. A similar use of these leaves was recorded earlier in the same area [53].

*Tanacetum balsamita *L. (Asteraceae). Alecost. Piedmont, N7, AB1, AB5. Local name: *Erba di San Pietro *[AB1],*Erba amara *[AB5]. Umbria, C3. Local name: *Erba della Madonna*. Cultivated and semi-domesticated/reverted to a wild status. The leaves are used to aromatize omelettes (especially on Easter Day [C3]), salads, and liqueurs. In AB1 they are fried with eggs, cheese, garlic cloves, and mallow leaves. Similar uses have been recorded in Northern Italy [41] and in Central Latium [46, 47].

*Tanacetum vulgare *L. (Asteraceae). Tansy. Piedmont, AB3. Local name: *Arquebuse*. The leaves are macerated in alcohol to make digestive liqueurs or used to aromatise omelettes. A couple of similar uses have been recorded in Italy [31].

*Tolpis quadriaristata *Biv. (Asteraceae). Umbrella milkwort. Wild. Sicily, S4. Local name: *Scaloredda*. The young leaves/whorls are collected during the winter and boiled in soups. They are thought to add a particular flavour to vegetable soups. A similar use has been recorded in Eastern Sicily [43].

## Conclusion

The data that we have presented here showed that gathering, processing and consuming wild food plants are still important activities in all the selected areas.

A few botanicals have been quoted and cited in multiple areas, demonstrating that there are important ethnobotanical contact points among the various Italian regions. One taxon (*Borago officinalis*) was among the most quoted taxa in both the Southern and the Northern Italian sites.

However, we observed that there were a few differences in the gathering and consumption of wild food plants between these areas. In the North, Rosaceae species prevailed, whereas in the South, taxa belonging to the Asteraceae, Brassicaceae, and Liliaceae s.l. families were most frequently cited. We proposed the hypothesis that these differences may be due to the likelihood that in Southern Italy the erosion of TK on wild vegetables is taking place more slowly, and also to the fact that Southern Italians probably have a higher appreciation of wild vegetables/green, which have often a strong or bitter taste.

Statistical analysis confirmed that there were major differences in the frequencies of quotation of the wild plants between Southern and Northern Italy, and that these can be ascribed only partially to ethnic/cultural issues. Additional crucial factors in the selection criteria of wild food plants could be also represented by recent socio-economic shifts, such us the increasing interest in local foods/"prodotti tipici" among middle-aged and young urbanised groups.

Finally, a brief remark on the few hypotheses that have been proposed in recent years by our research group and by others [9,54,55] – regarding the crucial role that the ethnicity/historical origin of human groups may play in determining the folk usages of botanicals: we feel that the proposed link between the persistence of culturally-specific linguistic labels in plant folk taxonomies and *specific unique ethnobotanical uses *should probably be evaluated and substantiated by more solid quantitative and statistical methods, and probably within more socio-anthropological oriented perspectives, rather than merely cognitive, anthropological ones: "universalistic" hypothesis regarding human selection criteria of plants for food and medicine, which have been proposed by other authors [22,39,56], could not be in fact verified in our comparative study.

## Authors' contributions

MPG, MC, NdV, AR, OC, FV, GA, MM, DA, MT, FL, IM, NC, TG, FD, AM, PR, CS, MB, RB, EB, AT, MG, LV, PC, ES, GA, EC and GB collected the data in the twenty-one sites and contributed to the discussion. AP analysed the data and drafted the theoretical framework for the discussion.
